# Spine-Prints: Transposing Brain Fingerprints to the Spinal Cord

**DOI:** 10.1101/2025.05.30.656545

**Published:** 2025-06-02

**Authors:** Ilaria Ricchi, Andrea Santoro, Nawal Kinany, Caroline Landelle, Ali Khatibi, Shahabeddin Vahdat, Julien Doyon, Robert L. Barry, Dimitri Van De Ville

**Affiliations:** 1 Neuro-X Institute, École Polytechnique Fédérale de Lausanne (EPFL), Geneva, Switzerland; 2 Department of Radiology and Medical Informatics, University of Geneva, Geneva, Switzerland; 3 CENTAI Institute, Turin, Italy; 4 Athinoula A. Martinos Center for Biomedical Imaging, Department of Radiology, Massachusetts General Hospital, Charlestown, Massachusetts, United States; 5 McConnell Brain Imaging Centre, Department of Neurology and Neurosurgery, Montreal Neurological Institute, McGill University, Montreal, QC, Canada; 6 Department of Psychology, University of Bath, Bath BA2 7AY, United Kingdom; 7 Department of Bioengineering, University of California Riverside, United States; 8 Harvard Medical School, Boston, Massachusetts, United States; 9 Harvard-Massachusetts Institute of Technology Health Sciences & Technology, Cambridge, Massachusetts, United States

**Keywords:** fMRI, functional connectivity, brain fingerprinting, spinal cord imaging, inter-subject variability

## Abstract

Functional connectivity (FC) patterns in the human brain form a reproducible, individual-specific “fingerprint” that allows reliable identification of the same participant across scans acquired over different sessions. While brain fingerprinting is robust across healthy individuals and neuroimaging modalities, little is known about whether the fingerprinting principle extends beyond the brain. Here, we used multiple spinal functional magnetic resonance imaging (fMRI) datasets acquired at different sites to examine whether a fingerprint can be revealed from FCs of the cervical region of the human spinal cord.

Our results demonstrate that the functional organisation of the cervical spinal cord also exhibits individual-specific properties, suggesting the potential existence of a spine-print within the same acquisition session.

This study provides the first evidence of a spinal cord connectivity fingerprint, underscoring the importance of considering a more comprehensive view of the entire central nervous system. Eventually, these spine-specific signatures could contribute to identifying individualized biomarkers of neuronal connectivity, with potential clinical applications in neurology and neurosurgery.

## 1. Introduction

Functional magnetic resonance imaging (fMRI) provides a non-invasive tool for capturing the spatiotemporal features of brain activity across individuals (McGonigle et al. 2000; Dubois and Adolphs 2016). For resting state, pairwise correlations between regionally-averaged time series define a functional connectivity (FC) profile that characterizes statistical dependencies between activity in different brain regions (Sporns, Tononi, and Kötter 2005; Fornito, Zalesky, and Bullmore 2016). While most of the early studies in neuroimaging have analyzed FC patterns at the group level to identify robust population trends (Margulies et al. 2016; Snyder and Raichle 2012), such approaches might overlook meaningful inter-individual differences. In the human brain, FC profiles have been shown to act as distinctive neural “fingerprints”. The pivotal study of (Finn et al. 2015) demonstrated that each individual’s FC pattern remains consistent across multiple sessions and conditions, allowing to reach up to 95% identification accuracy across 126 subjects at rest. In this context, the frontoparietal network emerged as being particularly distinctive, hence enabling accurate identification of individualized FC patterns, underscoring their potential for personalized neuroimaging applications. Over the past decade, a growing body of work has shown that the accuracy of brain fingerprinting depends on multiple factors, including scan length, the inter-scan interval (Amico and Goñi, 2018; Horien et al., 2019), and the presence of different clinical (Sorrentino et al. 2021; Stampacchia et al. 2022, 2021) or cognitive conditions (Luppi et al. 2023,2025; Tolle et al. 2024). Methodological advances have also improved performance. For instance, (Amico and Goñi 2018) examined approaches to enhance the distinctiveness of individual functional connectomes using dimensionality reduction based on principal component analysis (PCA). They quantified how consistently an individual’s FC profile could be recognized across different sessions, achieving near-perfect classification when only the most informative connections were retained.

Similarly, (Li, Wisner, and Atluri 2021) investigated optimal strategies for feature extraction in brain fingerprinting, by selecting the most relevant edges rather than the whole brain connections. In terms of the dynamic and temporal features of brain fingerprints, (Van De Ville et al. 2021) showed that identification is possible even at short time scales (*i.e*. in less than 30 seconds of fMRI activity), but that the strength fluctuates significantly over time. In a more recent study, (Ghaffari et al. 2025) demonstrated that the subcortical-cerebellum network had a substantial contribution in the static case, while playing a less prominent role in any of the dynamic fingerprints. Other studies have also investigated an individualized approach using Connectome Fingerprint (Tobyne et al. 2018), which is more focused on the prediction of individualized task activation. Building on this work, (Tripathi and Somers 2023) found that using a subject’s own FC generally resulted in higher prediction accuracy for their task activations compared to using connectivity from other individuals. In particular, FC patterns between the cerebral cortex and cerebellum carried crucial information for these predictions. A minor involvement of the cerebellum for the fingerprint accuracy was also found (Mantwill et al. 2022). Beyond fMRI, brain fingerprinting extends across various neuroimaging modalities, including electroencephalography (EEG) (Fraschini et al. 2016; W. Kong et al. 2019; Demuru and Fraschini 2020), functional near-infrared spectroscopy (fNIRS) (de Souza Rodrigues et al. 2019), and magnetoencephalography (MEG) (da Silva Castanheira et al. 2021; Sareen et al. 2021), highlighting the robustness of individual-specific connectivity patterns across different measurement techniques.

While brain functional connectivity has been widely studied, the same cannot be said for the spinal cord, despite its pivotal role in sensory and motor functions (for reviews Landelle et al. 2021, Kinany et al. 2022). However, the spinal cord is more than a pathway for relaying sensory and motor signals between the brain and the effectors. A large body of literature demonstrates that the spinal cord is also involved in a range of integrative and plastic processes, including learning (Grau 2014; Vahdat et al. 2015; Khatibi et al. 2022; Wolpaw 2007), pain modulation, and modulation of descending signal from supraspinal structures (Ossipov, Morimura, and Porreca 2014; Martucci and Mackey 2018; Nim et al. 2021). Furthermore, a series of pioneering studies (Khatibi et al. 2022; N. Kinany et al. 2023; Vahdat et al. 2015) investigating motor sequence learning with simultaneous human brain and spinal cord fMRI have provided *in vivo* evidence supporting learning-related plasticity within the spinal cord. Complementing this, more recent findings suggest that the human spinal cord is also involved in predictive processing, with prior knowledge influencing spinal responses to sensory stimuli as early as 13–16 milliseconds after stimulation (Stenner et al. 2025). These insights are further supported by neurophysiological evidence pointing to a complexity in spinal function beyond its classical sensorimotor role, including contributions to affective dimensions. For example, observing others in pain can evoke spinal responses similar to those triggered by first hand pain (Tinnermann, Büchel, and Haaker 2021). Moreover, local interneuronal circuits appear to support task learning, and computational models suggest that these circuits may self-organize through Hebbian learning (Enander, Loeb, and Jörntell 2022). Altogether, these processes may give rise to nuanced, individual-specific connectivity patterns in the spinal cord. Supporting this idea, test-retest experiments have shown that functional connectivity profiles remain stable across sessions, suggesting consistent and potentially unique signatures at the individual level—a prerequisite to probe the existence of a “spinal cord fingerprint”, or “spine-print” (Barry et al. 2016; Kaptan et al. 2023; Kowalczyk et al. 2024).

Inspired by brain fingerprinting and the spinal-cord reliability studies, herein we provide the first direct evidence supporting the existence of a spinal cord fingerprint, for which we coin the term “spine-print”. We investigate whether individual FC patterns could be reliably identified in the cervical spinal cord, akin to those observed in the brain. To test this, we computed identifiability scores from cervical spinal cord FC profiles using three independent datasets, one of which included simultaneous brain and spinal cord acquisitions. Following (Amico and Goñi 2018), we implemented a pipeline to extract identifiability metrics and assess the reliability of specific spinal connections, and successfully confirmed the existence of spine-prints including which functional connections were most distinctive across individuals. In addition to assessing spinal cord identifiability in isolation, we established the individual fingerprints of the brain, the spinal cord, and their interaction. These findings contribute to a growing body of work suggesting that the spinal cord, far from being a mere relay, may play a dynamic and individual-specific role in the human central nervous system.

## 2. Methods

### 2.1. Datasets

This study examines three distinct datasets: *Dataset 1* (partially published data from Vanderbilt University), which originally comprised 20 healthy volunteers (10 female, mean age 32±11 years). However, two female participants were excluded—one due to missing physiological recordings and the other because of a higher slice placement along the spinal cord, which complicated normalization to the template and reduced consistency in spinal level overlap across participants; *Dataset 2* from the Max Planck Institute in Leipzig, Germany that consists of 43 participants selected from a pool of 48 subjects in the publicly available OpenNeuro data (Kaptan et al. 2023): five participants were excluded (four due to missing physiological data and one due to a different number of acquired volumes); Dataset 3 (unpublished data with similar acquisition protocol to (Landelle et al. 2024)) includes 15 participants (7 female, 30±6 years old).

Participants in *Dataset 1* provided written informed consent under a protocol approved by the Vanderbilt University Institutional Review Board. These data were then analyzed under a protocol approved by the Mass General Brigham Institutional Review Board. *Dataset 2* was already published (Kaptan et al. 2023), having therefore ethical approval. *Dataset 3* study was approved by the ethic committee of the Institut universitaire de gériatrie de Montréal (#CERVN 17-18-20), which follows the policies of the Canadian Tri-Council Research Ethics Policy Statement and the principles expressed in the Declaration of Helsinki.

### 2.2. Data acquisition

#### Dataset 1

These data were acquired by R. Barry while at the Vanderbilt University Institute of Imaging Science. Experiments were performed on a Philips Achieva 3T scanner (Best, The Netherlands) with a dual-channel transmit body coil and the vendor’s 16-channel neurovascular coil (6 head, 4 neck, and 6 upper chest channels). Each scanning session began with a sagittal localizer to identify the general anatomy and location of the C3/C4 intervertebral disc. The imaging stack was then centered at this level, ensuring that all slices were perpendicular to the cord. The imaging stack covered vertebral levels C2-C5, roughly corresponding to the spinal nerve root levels C3-C6. High-resolution axial anatomical images were acquired using an averaged multi-echo gradient echo (mFFE) T2*-weighted sequence with the following parameters: field of view $(\text{FOV})=150\times 150{\;\text{mm}}^{2}$, acquired $\text{voxel}\;\text{size}=0.65\times 0.65\times 5{\text{mm}}^{3}$, interpolated $\text{voxel}\;\text{size}=0.29\times 0.29\times 5\;{\text{mm}}^{3}$, 12 slices, first TE = 7.20 ms, 4 additional echoes where ΔTE=8.83ms (5 echoes in total), repetition time (TR) = 700 ms, flip angle = 28°, sensitivity encoding (SENSE) = 2.0 (left-right), and number of acquisitions averaged = 2. Total acquisition time for the anatomical scan was 5 mins and 26 s. A saturation band was positioned anterior to the spinal cord to suppress signal from the mouth and throat. These anatomical data were presented in a previous publication measuring T2* in spinal cord gray matter (Barry and Smith 2019). Resting state fMRI data were acquired with identical slice placement using a 3D multi-shot gradient-echo sequence: volume acquisition time (VAT) = 2080 ms, TR/TE = 34/8 ms, flip angle = 8°, $\text{voxel}\;\text{size}=1\times 1\times 5\;{\text{mm}}^{3},\;12$ slices. The two separate 10-min (288 volumes each) were separated by a ~2-min period to give the subject a chance to rest. Respiratory and cardiac cycles throughout both runs were externally monitored and continuously recorded using a respiratory bellows and a pulse oximeter. These fMRI data were previously analyzed and presented in a conference abstract (Rangaprakash Deshpande & Barry Robert 2021).

#### Dataset 2

The second dataset, described in detail in (Kaptan et al. 2023), was acquired using a 3T Siemens Prisma MRI system equipped with a whole-body RF coil, a 64-channel head-and-neck coil, and a 32-channel spine-array coil. Participants were instructed to remain still, breathe normally, and avoid excessive swallowing. The dataset is part of a larger methodological project, focusing on two functional MRI acquisitions and one structural acquisition.

Functional MRI runs included 250 single-shot gradient-echo EPI volumes (VAT = TR × number of shots (1) = 2312 ms, 9.63 min of duration), covering the spinal cord from C2 to T1 with 24 slices (5 mm thickness) and a cross-section resolution of $1.0\times 1.0{\;\text{mm}}^{2}$. The acquisitions used z-shimming to counteract signal loss due to magnetic field inhomogeneities, with two runs differing in manual vs. automatic z-shim selection.

A high-resolution T2-weighted 3D sagittal SPACE sequence $\left(0.8\times 0.8\times 0.8{\;\text{mm}}^{3}\right)$ was acquired for registration. Peripheral physiological signals (respiration via a breathing belt and cardiac activity via ECG electrodes) were also recorded for physiological noise modeling.

#### Dataset 3

The last dataset was acquired by J. Doyon’s group. It is characterized by 2 functional runs back-to-back, also intervalled by a few minutes following similar acquisition parameters as Landelle et al. 2024. Data were acquired on a 3T MRI Scanner (Magnetom-Prisma, Siemens, Erlangen, Germany) with a 64 channel head and neck coil. The blood-oxygen-level-dependant (BOLD) images were acquired using a gradient-echo echo-planar imaging (EPI) sequence with the following parameters: VAT = TR = 1550 ms, TE = 23 ms, in-plane $\text{voxel}\;\text{resolution}=1.6\times 1.6{\;\text{mm}}^{2}$; slice thickness = 4 mm, FOV = 120 mm × 120 mm, flip angle = 70°; iPAT acceleration factor PE = 2, iPAT acceleration factor slice = 3. A total of 69 axial slices were acquired from the top head to C7/T1 vertebrae. Each resting-state run lasted 10 minutes and 35 seconds (230 volumes). Physiological recordings were acquired using a pulse sensor and a respiration belt (Siemens Physiology Monitoring Unit).

Brain/spinal cord structural images were acquired using a high-resolution T1-weighted anatomical image in the sagittal direction (MPRAGE sequence: TR/TE = 2300/3.3 ms, $\text{voxel}\;\text{size}=1.3\times 1.3\times 1.3\;{\text{mm}}^{3},\;\text{FOV}=365\times 375{\;\text{mm}}^{2}$, flip angle = 9°). In total, 288 slices were acquired, covering the top of the head to the upper thoracic regions T2-weighted images were acquired, spanning from the top of the cerebellum to the upper thoracic region (approximately at T1), thereby encompassing the entirety of the cervical spinal cord. These images were collected in the transverse orientation with the following parameters: TR = 33 ms; TE = 14 ms; FOV = 211 mm × 249 mm; flip angle = 5°; in-plane voxel resolution = 0.35 mm × 0.35 mm, slice thickness = 2 mm.

### 2.3. Data preprocessing

We applied an in-house preprocessing pipeline for the first two spinal cord datasets publicly available on github (https://github.com/MIPLabCH/SC-Preprocessing.git), while the third brain/spinal cord dataset was preprocessed using (Landelle et al. 2024) preprocessing pipeline. The two pipelines include comparable preprocessing steps for the spinal cord; see (Landelle et al. 2024; Ricchi, Kinany, and Van De Ville 2024) for reference, no spatial smoothing was applied.

#### 2.3.1. Preprocessing of Datasets 1 & 2

The spinal cord functional and structural images were pre-processed using Python (version 3.9.19), with the nilearn library (version 0.9.1) falling under the umbrella of scikit-learn (version 0.24.2), FMRIB Software Library (FSL; version 5.0), and Spinal Cord Toolbox (SCT; version 5.3.0; (De Leener et al. 2017)). The following preprocessing steps were performed: i) slice-timing correction (FSL, ‘*slicetimer*’), ii) motion correction using slice-wise realignment and spine interpolation (with SCT, ‘*sct_fmri_moco*’), iii) segmentation of functional and structural images (with ‘*sct_deepseg*’, followed by manual correction), iv) time series denoising (see details in the next paragraph), v) registration of functional images to anatomical images, and finally vi) coregistration of functional images to anatomical images, and then, to the PAM50 template (with SCT, ‘*sct_register*’ multimodal and ‘*sct_register_to_template*’ with nearest neighbor interpolation). Additionally, we assessed the data quality of the three datasets by obtaining voxelwise tSNR values with the SCT’s function ‘*sct_fmri_compute_tsnr*’ (De Leener et al. 2017), which computes each voxel’s temporal mean and divides it by its standard deviation.

#### 2.3.2. Time series denoising of Datasets 1 & 2

The time denoising series denoising follows the same procedure as previous spinal fMRI (Kinany et al. 2024; Landelle et al. 2024; Ricchi, Kinany, and Van De Ville 2024; Landelle et al. 2023) relying on the ‘*clean_img*’ function from the nilearn library, This approach enable the removal of the noise confounds orthogonally to the temporal filter. Specifically, confounds and the band-pass temporal filter (cut-off frequencies: 0.01 Hz and 0.13 Hz) were projected onto the same orthogonal space, following the methodology outlined in (Lindquist et al. 2019), instead of being applied sequentially.

Physiological noise correction was performed using a model-based approach inspired by RETROICOR (RETROspective Image CORrection; (Glover, Li, and Ress 2000)), which models physiological signals as quasi-periodic and maps their phases onto each image volume using a Fourier series expansion. To implement this, we used FSL’s Physiological Noise Modeling (PNM) tool to generate nuisance regressors from cardiac, respiratory, and cerebrospinal fluid (CSF) signals. Cardiac peaks were identified using the ‘*scipy.signal.find_peaks*’ function (Virtanen et al. 2020), followed by manual verification to ensure detection accuracy.

We followed established guidelines for physiological noise correction in spinal cord fMRI (Y. Kong et al. 2012). Both cardiac and respiratory regressors were modeled with an order of 4, including the fundamental frequency and the first three harmonics. Their interaction was modeled up to second order, producing a total of 32 regressors on a slice-by-slice basis. Additionally, a CSF regressor was derived by averaging the signal from the 10% most variable CSF voxels. Each of these regressors was applied independently per slice using the ‘*clean_img*’ function to account for the anatomical and physiological specificity of spinal cord data.

#### 2.3.3. Preprocessing and denoising of Dataset 3

As this dataset includes simultaneous brain and spinal cord acquisition, we adapted the previously described approach accordingly. For each participant, all the functional and structural images were pre-processed separately for the brain and the spinal cord using the Spinal Cord Toolbox (SCT; version 5.6), FSL, SPM, and in-house Python and MATLAB programs. First, T1w images were cropped to separate the brain and spinal cord. Spinal cord segmentation was carried out using SCT (sct_propseg) and manually corrected when necessary. The brain structural images were segmented using Cat-12 toolbox (SPM) into gray matter (GM), white matter (WM), and CSF.

The spinal cord volumes of each functional run were averaged, and the centerline of the cord was automatically extracted from the mean image (or drawn manually when needed). A cylindrical coarse mask with a diameter of 15 mm along this centerline was drawn and further used to exclude regions outside the spinal cord from the motion correction procedure, as those regions may move independently from the cord.

Motion correction was performed using the first volume as the target image and resulted in the motion corrected time series (‘*sct_fmri_moco*’ from SCT). Moco was carried out using slice-wise translations in the axial plane with spline registration. The resulting mean image of the motion corrected images was used for segmentation of the cord using propseg (manual correction was done when necessary). The mean moco image was warped into the PAM50 space using the concatenated warping field obtained at the anat step (i.e., from T1w to T2w to PAM50 space) to initialize the registration (with SCT, ‘*sct_register_multimodal*’).

As for the brain volumes, they undergo a standard preprocessing (Power et al. 2014, 2012) using FSL ‘*bet*’ function for estimating the masks, FSL ‘*mcflirt*’ to apply motion correction, and coregistering the functional images to T1w and the MNI template using SPM12.

For each participant, the nuisance regressors were modeled to account for physiological noise (Tapas PhysiO toolbox, an SP extension (Kasper et al. 2017)). Peripheral physiological recordings (heart rate and respiration) using the RETROspective Image CORrection (RETROICOR) procedure (Glover et al. 2000). Specifically, four respiratory, three cardiac harmonics were modelled, and one multiplicative term for the interaction between the two (18 regressors in total, (Kinany et al. 2024; Landelle et al. 2024)). CompCor approach (Behzadi et al. 2007) was used to identify non-neural fluctuations in the unsmoothed brain or CSF signal by extracting the first 12 principal components for the brain and the first 5 for the spinal cord. These nuisance regressors were complemented with the two spinal cord motion parameters (x and y), the six brain motion parameters and motion outliers.

Similar to dataset 1 and 2 the removal of the noise confounds was based on a projection onto the orthogonal of the fMRI time-series space and was applied orthogonally to the band-pass temporal filter (0.01–0.13 Hz) using the Nilearn toolbox (‘*clean_img*’ function (Lindquist et al. 2019)).

#### 2.3.4. Parcelled time series

##### Spinal cord

**Figure 1.**
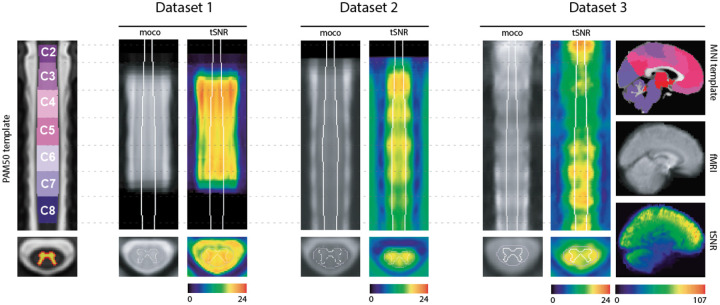
Datasets overview. Left panel: coronal view of the PAM50 template (y=70) with spinal level annotations (C2-C8) and the gray matter probability mask on the axial view. The three datasets are shown sequentially with the mean motion-corrected fMRI and the tSNR maps. Dataset 3 additionally includes whole-brain coverage, with the brain data shown alongside the MNI template of 2mm resolution in the sagittal plane (x=45).

We defined a parcellation scheme consisting of 14 spinal cord regions in the cross-section characterized by 6 bilateral regions of the gray matter (intermediate zone (*iz*), ventral (*vh*) and dorsal horns (*dh*)) and 8 bilateral white matter regions (spinal lemniscus (SL), cortico-spinal tract (*cst*), fasciculus cuneatus (*fc*) and gracilis (f*g*)). These anatomical regions were delineated according to the atlas of the Spinal Cord Toolbox (De Leener et al. 2017). The number of spinal levels included is determined by the acquisition coverage (Figure 1) and the overlap among participants. Levels at the extremities were cropped in some participants; thus, to maintain consistency, only levels fully overlapping across all participants are considered. Specifically, *Dataset 1* included 3 spinal levels (C4–C6), resulting in a parcellation of 42 regions of interest (ROIs); *Dataset 2* comprised 5 fully overlapping spinal levels (C4–C8), yielding a dimensionality of 70 parcelled time series; and Dataset 3 spanned spinal levels C2–C8, covering a total of 7 spinal levels and resulting in 98 ROIs.

The voxel time series from the registered, denoised functional images were averaged using a robust mean, considering only time points between the 5th and 95th percentiles. In the paper we will refer to the number of spinal cord ROIs with $N_{sc}$.

##### Brain

For *Dataset 3*, we also extracted parcelled brain time series following the same procedure: computing the robust mean across voxels within each of the 100 ROIs defined by the Schaefer’s atlas (Schaefer et al. 2018) and complemented by 19 subcortical areas (Seitzman et al. 2020), resulting with a parcellation of $N_{\text{br}}=119$.

### 2.4. Fingerprinting

#### 2.4.1. Methodology

We follow the steps proposed in earlier work at the brain level (Amico and Goñi 2018). Let us assume $N_{s}$ participants for which $F_{i}^{r}$ denotes the FC matrix of run r for participant i. Each matrix $F_{i}^{r}$ has a dimensionality of N×N, where N indicates the number of ROIs (which can be $N_{\text{sc}}$ or $N_{\text{br}}$, depending on whether spinal cord or brain regions are considered). The concept of fingerprinting centers on the ability to identify individuals based on these FC patterns; i.e., by comparing an individual’s FC matrix from one run against those from all participants, including another run from the same subject (Finn et al. 2015; Amico and Goñi 2018). In this framework, two runs are available per subject (test-retest). To enable comparison, the upper triangular part of each $F_{i}^{r}$ is unfolded to a vector $c_{i}^{\left(r\right)}$ of dimension M=N(N-1)/2, to which Pearson correlation is applied. This results in the so-called identifiability matrix I, of size $N_{s}\times N_{s}$, which is asymmetric. The diagonal elements I[i,i] reflect self-similarity between the two runs of the same participant, while the off-diagonal elements I[i,j] (with i≠j) capture between-participant similarities. Let $I_{\text{self}}$ be the average of the diagonal elements, and $I_{\text{others}}$ the average of the cross-individuals similarities. We can now define a fingerprinting performance metric (‘*Idiff*’) as the difference between these values $\left(I_{\text{self}}-I_{\text{others}}\right)$. Moreover, following also (Amico and Goñi 2018), we explored the PCA of the FC profiles to maximize *Idiff* by applying dimensionality reduction (see supplementary material for details).

To further quantify the strength of the fingerprinting effect, we calculated Cohen’s D (Cohen 1992) as a standardized measure of the difference between two means ($I_{\text{self}}$ and $I_{\text{others}}$ in particular), reflecting how large this difference is relative to the variability within the data. Let $\sigma_{\text{self}}^{2}$ be the variance of the self-similarities and $\sigma_{\text{others}}^{2}$ the variance of the off-diagonal elements, then the effect size is defined as follow:

(1) $$d=\frac{I_{\text{self}}-I_{\text{others}}}{\sqrt{\frac{\sigma_{\text{self}}^{2}+\sigma_{\text{other}}^{2}}{2}}}.$$

A higher Cohen’s D value indicates a greater separation (in standard deviation units) between individuals, thus stronger identifiability.

Following (Finn et al. 2015), we also calculated the success rate as the number of participants that could be identified. A participant was considered correctly identified if the highest correlation in their row of the identifiability matrix appeared on the diagonal. This indicates that their FC profile from the first run was more similar to their own profile than to that of any other participant from the second run. We also propose a more lenient measure by computing the participants’ identification accuracy based on whether the correct participant was within the top 2, 3, 4, or 5 highest correlation values.

To assess which FC patterns contributed most reliably to individual identifiability, we used the one-way random model for the intraclass correlation coefficient that is known as ICC(1,1) (McGraw and Wong, 1996). Specifically, this metric measures the absolute agreement of repeated measurements for each connection across individuals:

(2) $$\text{ICC}\left(1,1\right)=\frac{MS_{R}-MS_{W}}{MS_{R}+\left(k-1\right)MS_{W}},$$

where k=2 (number of runs), $MS_{R}$ is the mean square between individuals, and $MS_{W}$ is the within-participant (error) variance. This computation was repeated independently for each edge, yielding a vector of ICC values, which were then mapped back to the N×N space to obtain the full ICC matrix. This method provides a fine-grained identifiability map of which FC edges are most stable across runs, reflecting their test-retest reliability. To have an overall overview of these reliable edges in the context of the spinal cord, we averaged the ICC matrices across datasets and all levels. For the cervical spinal cord, we recall that the number of ROIs is 14 in the cross-section (i.e., bilaterally for gray matter: *dh*, *vh*, *iz*; and for white matter: *cst*, *fc*, *fg*, *sl*). Moreover, we examined the ICC matrices after thresholding at the 95th percentile to retain only the most reliable edges. To shift the focus from the connections to region-based reliability, we computed the nodal strength of this filtered matrix by summing the values across one axis, providing a measure of reliability for each ROIs rather than for specific connections.

#### 2.4.2. Investigating brain and spinal cord time courses

To further explore Dataset 3, we applied the fingerprinting pipeline to the full FOV including brain and cervical spinal cord, and looked at three FC profiles: brain-only, spine-only, and brain-spine interaction. In addition, we examined the relationship between brain and spinal time series using a simple linear regression framework (equation 3) in two directions: (i) modeling spinal cord activity (denoted as Y) using brain signals (X) as predictors, and (ii) modeling brain activity (Y) using spinal cord signals (X) predictors.

Since the imaging was acquired simultaneously, the time axis is shared between the dependent (Y) and independent variable (X). Both matrices have dimensions N×T, where N corresponds to the number of ROIs − either $N_{\text{sc}}$ for spinal cord or $N_{\text{br}}$ for brain − and T is the number of time points. As a result, the regression coefficients β have also different dimensionality depending on the direction of modeling. We denote the brain-to-spine model coefficients as $\beta_{\text{sc}-\text{br}}$ (of size $N_{\text{sc}}\times N_{\text{br}}$), and the reverse model as $\beta_{\text{br}-\text{sc}}$ (of size $N_{\text{br}}\times N_{\text{sc}}$).

The linear regression is defined as:

(3) Y=X⋅β+ϵ,

where $\hat{Y}=X\cdot \beta$ are the predicted values and $\epsilon =Y-\hat{Y}$ represents the residuals. In the first, model the residuals $\epsilon_{\text{sc}-\text{br}}$ capture the portion of the spinal signal not explained by the brain activity and dimensionality $N_{\text{sc}}\times T$. In the second model, the residuals $\epsilon_{\text{br}-\text{sc}}$ represent the portion of the brain signal not explained by the spine and have dimensionality of $N_{\text{br}}\times T$. We then used these residuals to compute new FC profiles that reflect variance not accounted for by the other region.

## Results

### 3.1 Data quality and overview

In Figure 1, we present an overview of the three datasets analyzed in this study together with the template spaces that were employed: PAM50 for spinal cord data (left) and MNI for brain data (right, shown alongside Dataset 3, which covers the brain). The ROIs in the brain were defined using the Schaefer-100, complemented by 19 subcortical areas, and are visualized on the MNI template. For the spinal cord, only spinal levels C2-C8 are shown on the PAM50 template, reflecting the coverage range of the spinal cord acquisitions across datasets. Mean fMRI images and tSNR maps of the first run are also provided for each dataset. In the spinal cord, tSNR values reach a maximum mean value of 22.6 in *Dataset 3*, followed by 21.3 for *Datasets 1*, while in *Dataset 2*, the maximum value is 19 (within the cord mask). By comparison, brain tSNR values are substantially higher, reaching up to 107.

### 3.2. Fingerprint of the central nervous system

**Figure 2.**
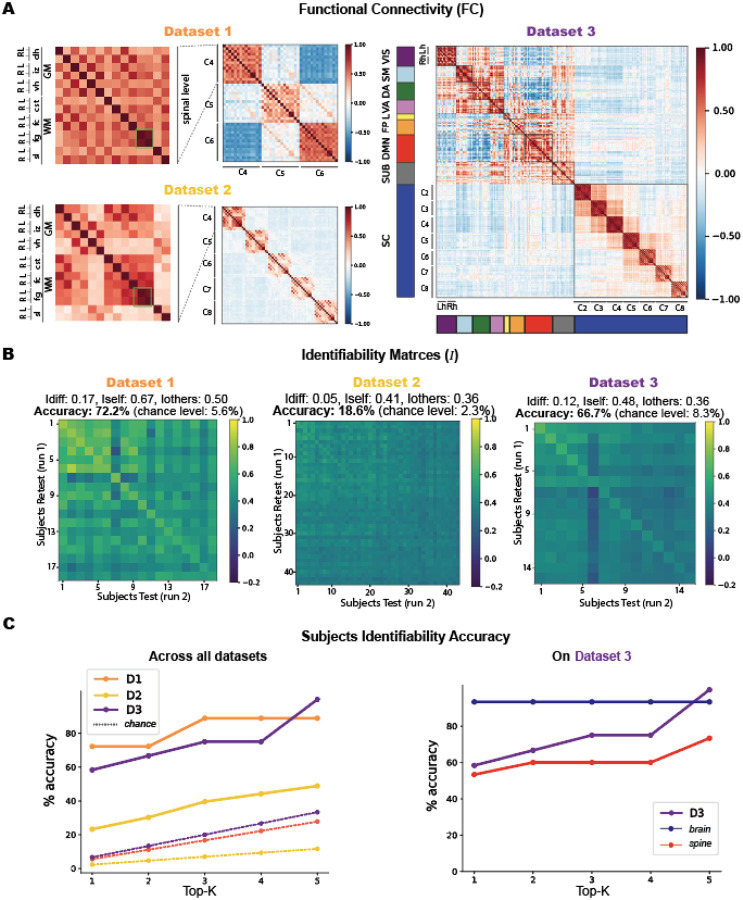
Overview of functional connectivity (FC) matrices and identifiability results for the three datasets. A. FC matrices of the first run from each dataset. The most-left matrix is a zoomed-in view of the spinal level C4 for both *Dataset 1* and *2* to show the labeling and sorting of the spinal cord ROIs used for all the spinal levels in all matrices. On the right *Dataset 3* FC displays the brain ROIs with Yeo’s sorting of the 7 functional networks (VIS, SM, DA, VA, L, FP, DMN), followed by the subcortical regions (SUB) and the 7 spinal levels. The brain functional networks have the left hemisphere first (Lh) and the right (Rh) after. B. The second row shows the corresponding identifiability matrices for each dataset, along with the reached scores and accuracies. C. On the left, a plot illustrates participants identifiability accuracy as a function of the top-K (from 1 to 5) highest correlation values across the three datasets. On the right, a detailed plot for *Dataset 3* shows identifiability accuracy as a function of top-K values, with separate curves for brain-only and spine-only data.

In Figure 2, we display the FC (A) and identifiability matrices (B) derived from the three datasets. The zoomed-in panels in (A) highlight the anatomical organization of the ROIs in the spinal cord, with gray and white matter regions alternating from left to right—a sorting scheme that remains consistent throughout the paper whenever spinal levels are referenced. Brain regions, on the other hand, are sorted according to the Yeo functional networks (Yeo et al. 2011), concatenating left and right hemispheres (Lh, Rh) within each network.

Across all datasets, spinal cord connectivity reveals a strong bilateral (left-right) correlation, within the gray matter. The most prominent connectivity involves the **intermediate zone (*iz*)**, which exhibits strong correlations with the **ventral horns (*vh*)**, followed by weaker but consistent connections with the **dorsal horns (*dh*)**. Beyond the GM, we also observe bilateral connectivity patterns in the white matter. Among these, the **fasciculus gracilis (*fg*)** stands out as the only ROI that consistently exhibits a distinct and highly bilateral connectivity pattern across all spinal levels and datasets, as highlighted in the green boxes of Figure 2A, and the **fasciculus cuneatus (*fc*)** connectivity with some gray regions.

In *Dataset 1*, the highest correlation is observed in the bilateral *fg*, reaching 0.69. This is followed by the *iz-vh* connectivity, with values of 0.50 on the right and 0.49 on the left side. The *fc-dh* also exhibits strong connectivity, with 0.47 on the left and 0.46 on the right. The IZ connects with the fasciculus cuneatus (*fc*) at 0.43 on the right and 0.41 on the left side. *Fg* and *fc* show bilateral correlations of 0.40. Within the gray matter, the *dh-iz* connection reaches 0.38 on the right and 0.34 on the left, while *dh-iz* connectivity is slightly lower, at 0.27 (right) and 0.24 (left). Finally, bilateral connectivity within the ventral horns (VL–VR) is 0.23, and within the dorsal horns (DL–DR) it is 0.14.

*Dataset 2* shows a similar profile. Bilateral *fg* connectivity remains the strongest, with a correlation of 0.66. This is followed by *dh–fc* connectivity, with 0.47 on the left and 0.45 on the right. The *iz-vh* connections show values of 0.42 (left) and 0.41 (right), while *fg-fc* correlations are consistent at 0.39 on both sides. *Dh-iz* connections are slightly lower, at 0.28 (right) and 0.27 (left). Interestingly, VL–VR and DL–DR correlations are 0.13 and 0.12, respectively, slightly exceeding the horn-to-horn connectivity observed elsewhere, which typically remains below 0.1.

In *Dataset 3*, similar trends persist, but with overall stronger correlations. Bilateral *fg* connectivity reaches its highest value at 0.83. The *iz-vh* and *dh-fc* connections also show very strong bilateral correlations, both around 0.80. The *fg-fc* correlation remains high at 0.75 bilaterally. In this dataset, the **corticospinal tract (*cst*)** also displays strong connectivity with the dorsal horns, reaching 0.69 on the right and 0.68 on the left side. Gray matter connections follow with *dh-iz* at 0.61 and *dh-vh* at 0.49 on both sides. Finally, the ventral horn bilateral correlation (VL–VR) reaches 0.33, and the dorsal horns (DL–DR) show a correlation of 0.27.

To assess the consistency of these subject-averaged correlations, we computed the identifiability matrix *(I)*, which quantifies the extent to which each participant’s FC profile is distinct within the dataset across two runs (Figure 2B). We recall that I is an $N_{s}\times N_{s}$ matrix, where $N_{s}$ is the number of participants in a dataset. These matrices exhibit a clear diagonal pattern, indicating that each participant’s FC profile is more similar to their own than to anyone else’s. The maximum correlation values observed on I are 0.85 for *Dataset 1*, 0.6 for *Dataset 2*, and 0.68 for *Dataset 3*. *Dataset 1* reaches an Idiff=0.17 with a subject identification accuracy of 72.2% (13 participants were correctly identified over 18, chance level = 5.6%). Dataset 2 reaches an Idiff=0.05, accuracy = 18.6% (8 participants correctly identified over 43, chance level = 2.3%). *Dataset 3* Idiff=0.12, accuracy = 66.7% (10/15 correctly identified, chance level = 8.3%). Cohen’s D effect sizes of *Iself* and *Iothers* provide a measure of the magnitude of their difference. That is, *Datasets 1 and 3* exhibit very large effect sizes with 1.40 and 1.43, respectively. While Cohen’s D values of the brain-only were 1.87, of the spine only 0.96, and their interaction (brain-spine) 0.93. Despite the relatively low *Idiff* values and accuracy in *Dataset 2*, it still shows a medium effect size (Cohen’s D = 0.7). Typically, effect sizes are considered small between 0.2–0.4, medium between 0.5–0.8, and large above 0.8.

The accuracies across the three datasets display an upward trend (Figure 2C, left column) when considering whether each participant was among the top 1 to 5 highest correlation values. In *Dataset 3*, when analyzing brain and spine data separately, the accuracy stays constant for the brain, while it shows an increase for the spine.

### 3.3. Reliability of the fingerprint

**Figure 3.**
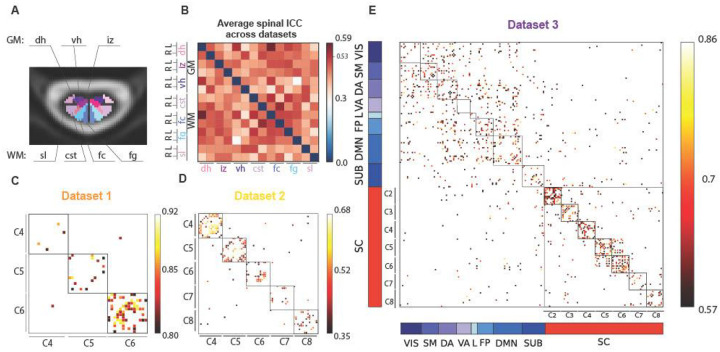
ICC results. (A) Spinal cord ROIs displayed in a cross section of the spinal cord (gray matter (GM) regions: *dh* = dorsal horns, *iz* = intermediate zone, *vh* = ventral horns, white matter (WM) regions: *sl* = spinal lemniscus, *cst* = cortico-spinal tract, *fc* = fasciculus cuneatus, *fg* = fasciculus gracilis). (B) Average ICC matrix across all spinal levels and datasets, indicating the 95th percentile in the colorbar (0.526). (C-D-E), namely, Datasets 1,2, and 3. (E) For Dataset 3, brain ROIs are sorted according to the Yeo functional networks (Yeo et al. 2011) (VIS = visual, SM = somatomotor, DA = dorsal attention, VA = ventral attention, L = limbic, FP = fronto-parietal, DMN = default mode network, SUB = subcortical). The color bar values are adjusted according to the distribution of the values, reporting the 95th percentiles of each dataset’s ICC as minimum to show a filtered ICC matrix.

The intraclass correlation coefficient (ICC) was used to evaluate the reliability of FC patterns across the two runs. To summarize spinal cord ROI reliability, we computed the mean ICC values across cervical levels for the three datasets (Figure 3B). According to standard guidelines (Hallgren 2012; Cicchetti and Sparrow 1981), ICC values are interpreted as: poor (<0.4), fair (0.4–0.59), good (0.6–0.74), and excellent (≥0.75).

Across all datasets, the most reliable connection in the spinal cord—above the 95th percentile threshold (ICC = 0.526)—was observed between the left dorsal GM horn and the left fasciculus cuneatus (ICC = 0.586), followed by *cst-fc* right connection (ICC = 0.56), and the left connections of the ventral GM horns and dorsal GM horns (ICC = 0.54), both falling within the “fair” reliability range.

Looking at each dataset individually: (i) *Dataset 1* showed excellent ICC values, particularly at the C6 level. When averaging ICC values across the three spinal levels to obtain nodal strength per ROI, the left *fc-dh* connectivity had the highest average ICC (0.81), followed by the bilateral *fc* with 0.78, (Figure 3C). *Dataset 2* exhibited overall lower ICC values, with the 95th percentile at 0.41 and a maximum ICC of 0.45 (interpreted as “fair”). Here too, nodal strength was highest for the left *fc-dh* connectivity (0.45), followed by the *fg-dh* on the left side, and lowest for the bilateral *cst-fc* (0.41) (Figure 3D). *Dataset 3* had higher reliability overall, with ICC values above 0.57 (95th percentile), and a maximum ICC of 0.86 (Figure 3E). In the brain, the most reliable connection (0.86) was the left salient ventral attention (VA) with the left DMN prefrontal cortex 5 (DMN) and the lowest ICC reached 0.63 with the left visual network 9 (VIS) and the right somatomotor 8. In the spinal cord, the most reliable connection was right *fc* with right spinal lemniscus (*sl*) with a good ICC of 0.6 and the lowest (0.57) was between *fc* and *vh* right.

Considering the nodal strength as mean values across the ICC matrix along one axis, in the brain, the values were relatively consistent across regions, averaging around 0.68, with the visual network and subcortical regions showing the lowest value (0.64), and default mode network (DMN) and limbic network (L) with 0.7. Interestingly in all three datasets, the spinal cord exhibit the strongest nodal strength for fasciculus cuneatus (*fc*), followed by fasciculus gracilis (*fg*) and dorsal horns (*dh*), while the lowest values differ in the three datasets: *Dataset 1* smallest nodal strength was found in the ventral horns, in *Dataset 2* it was the intermediate zone, and the *cst* for *Dataset 3*.

### 3.4. Brain and spine

**Figure 4.**
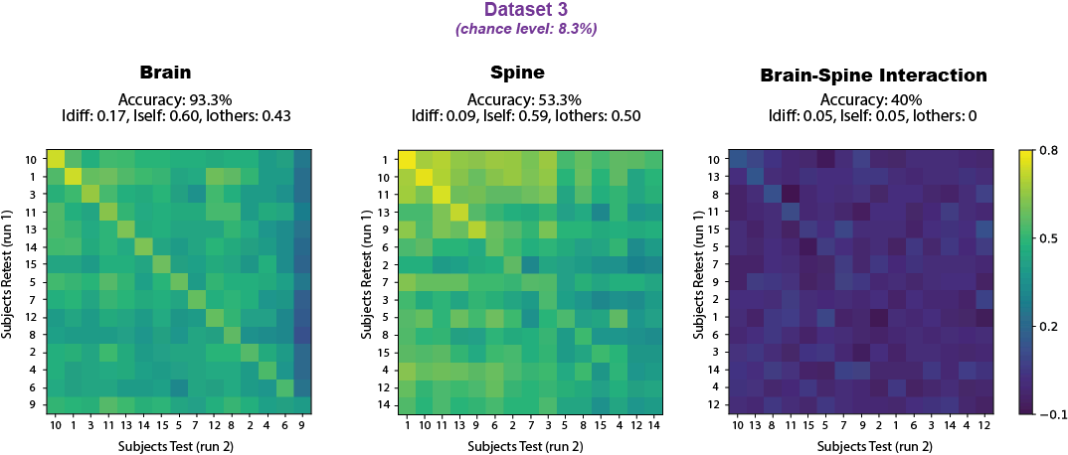
Dataset 3 fingerprinting using brain-only, spine-only, and brain–spine interaction as inputs. The identifiability matrices reflect the correlations across the participants’ runs, with participants’ IDs sorted by highest correlation in each case, leading to differing axis labels across matrices. Identification accuracies (chance level = 8.3%) are 93.3% for brain-only, 53.3% for spine-only, and 40% for the interaction.

We conducted a more in-depth analysis of Dataset 3, focusing on functional connections within the brain, the spinal cord, and between the two. In Figure 4, the identifiability matrices for each of these components are shown, along with their corresponding accuracy and identifiability scores. The brain-only matrix achieved the highest accuracy at 93.3%, with 14 out of 15 participants correctly identified. The spinal cord followed with 53.3% accuracy (8 correctly identified out of 15), while the brain-spine interaction block yielded an accuracy of 40% (6 out of 15).

Participants IDs in the matrices were sorted by decreasing correlation values within each component (brain-only, spine-only, and brain–spine), and, therefore, do not align across the three matrices. Nevertheless, certain individuals with high identifiability in the brain also performed well in the spine and brain–spine matrices; e.g., participants 1, 10, and 11. All three matrices exhibit a visible diagonal trend in the identifiability pattern, which is most pronounced in the brain matrix. For further details, the reader is addressed to Supplementary material.

We examined the residual correlations from linear regression models to assess their impact on subject identifiability. In the first model, where spinal cord time series served as the dependent variable (Y) and brain time series as the predictor (X), the residuals—representing spinal cord activity not linearly explained by brain activity—yielded an *Idiff* score of 0.07, with only 2 out of 15 participants correctly identified (accuracy = 13.3%). Conversely, in the inverse model, with brain time series as Y and spinal cord time series as X, the residuals—indicating brain activity unexplained by spinal cord signals—produced a higher *Idiff* of 0.12 and improved identification, correctly identifying 8 out of 15 individuals (accuracy = 53.3%).

## 3. Discussion

Our findings demonstrate that a reliable and individually distinctive FC pattern from the (cervical) spinal cord – termed “spine-print” – can be identified across individuals in three different datasets. Despite differences in acquisition protocols and slight variations in preprocessing, participants’ identification remains well above chance, highlighting the feasibility and robustness of the spine-print. This suggests that, much like the brain, the spinal cord exhibits participant-specific FC that is reliably detectable.

A closer inspection of the spine-prints and the regions with the highest reliability reveals that the most consistent areas—those with the highest nodal strength—were the sensory regions, rather than the ventral regions where motor neurons reside. This could suggest that the sensory functions processed by the cord might be more subject-specific, while motor functions at rest are more uniform across individuals. In Dataset 3, we also assessed the identifiability scores of the sensory-motor (SM) network alone in relation to the spinal cord, allowing for a fair comparison between the two. Interestingly, when the SM network and spinal cord regions were combined, the identifiability scores increased notably (see Supplementary Material, Fig. S4).

In terms of correlations, the bilateral connection of the fasciculus gracilis (*fg*) emerged as the strongest across all three datasets. The *fg* carries fine touch, vibration, and proprioceptive afferents from the lower limbs and trunk through heavily myelinated axons. Sensory input from bilaterally symmetric body regions may lead to similar fluctuation patterns in both the left and right fasciculi, particularly during rest. However, it is important to acknowledge that many of the strong bilateral correlations observed across the datasets could be largely influenced by partial volume effects, given the small size and close proximity of these regions. Additionally, the findings in the dorsal areas could also be affected by the presence of the dorsal vein, potentially confounding the interpretation of BOLD signals in these regions. While the interpretation of BOLD signals in the white matter remains debated, there is growing evidence that white matter has sufficient vascularization to support hemodynamic changes. Several studies have investigated the reliability of functional information captured in the cerebral white matter (Wu et al. 2017; Peer et al. 2017; Huang et al. 2020; Ding et al. 2018; Gawryluk, Mazerolle, and D’Arcy 2014; Gore et al. 2019). White matter is largely composed of glial cells, which regulate homeostasis and support myelinated axons, and their metabolic processes can influence local blood flow and presumably influence the oxygenation demand of the WM. These non-neuronal fluctuations may explain the distinct slower dynamics of BOLD signals in white matter compared to gray matter. Recognizing these factors is essential for accurate interpretation of spinal cord fMRI results (Sengupta et al. 2024; Paquette, Piché, and Leblond 2021). In line with this, dynamic functional connectivity approaches have revealed functional components in the spinal cord white matter during rest, as shown by (Kinany et al. 2020).

Importantly, an individual-specific signature could be primarily shaped by anatomical features. Prior studies have shown that spinal cord functional patterns are closely aligned with its structural organization (Kinany et al. 2020; Sengupta et al. 2021; Ricchi, Kinany, and Van De Ville 2024), supporting the notion that inter-individual anatomical variability may give rise to detectable functional differences.

However, while this spine-print exists, its distinctiveness is notably weaker compared to that of the brain. This difference raises important questions about the factors influencing the strength of individual signatures in different parts of the central nervous system, which we discuss next.

One such factor could be the relative granularity of the parcellation used to define ROIs. Notably, the number of ROIs in Dataset 3 was kept relatively comparable between the brain and the spinal cord: the brain was divided into 119 ROIs, while the spinal cord included 98, derived from 14 cross-sectional regions across 7 spinal levels. Yet, despite this similarity in dimensionality, the physical sizes of these structures differ drastically (Parent and Carpenter 1996; Sherman, Nassaux, and Citrin 1990). Their volumes are $1200-1400{\;\text{cm}}^{3}$ and about $11\;{\text{cm}}^{3}$, respectively, considering 8–10 cm of length for C1-C7 and a cross-section area of $1-1.5{\;\text{cm}}^{2}$. This results in a volume ratio of ~118. The ROIs from the atlases used for each are on average ~227 times smaller for the spine than for the brain. As a result, regional signal averages in the spinal cord are expected to be inherently noisier, especially given the lower tSNR typically observed in spinal fMRI. These factors together help explain the comparatively lower fingerprinting performance observed in the spinal cord relative to the brain.

Beyond size and signal quality, brain and spine also have different functional roles. For brain fingerprinting, the regions that are most specific for an individual are those serving higher-order cognitive functions, while sensory regions provide less unique information (Amico and Goñi 2018; Finn et al. 2015; Lu et al. 2024; Griffa et al. 2022; Jo et al. 2021). The latter are evolutionarily more preserved, and, given that the spinal cord is an even more ancient structure, it might therefore be presumed to exhibit limited individual variability and, consequently, a diminished capacity for supporting a distinct “spine-print”. Yet, despite these expectations, our results reveal a measurable degree of individual specificity in spinal cord FC. While this spine-print may partly reflect intrinsic functional properties of the spinal cord, it may also be shaped by brain activity. This is supported by our regression analyses assessing the dependency between brain and spinal cord time courses. When brain activity was regressed out of the spinal cord signals—yielding residuals used for fingerprinting—the identifiability scores dropped substantially. In contrast, removing spinal cord activity from the brain signals had little effect: the identifiability score (*Idiff*) remained high at 0.12, identical to the original Idiff obtained from the full dataset. Notably, using only the brain time series resulted in an even higher *Idiff* score of 0.17. These findings suggest that the brain may act as the primary driver of spinal cord activity patterns relevant for fingerprinting, and that the spine-print may, in part, reflect an echo of brain-derived individuality.

## 4. Conclusion and outlook

In this study, we demonstrate the feasibility of identifying an individual-specific functional signature in the spinal cord—a “spine-print”—across two runs within the same scanning session. This finding supports the notion that participant-level identifiability is not exclusive to the brain and can extend to spinal cord activity. Interestingly, brain regions involved in sensorimotor functions—those more directly related to spinal cord activity—tend to exhibit lower identifiability scores compared to higher-order cognitive regions such as the prefrontal and frontoparietal cortex (Amico and Goñi 2018; Finn et al. 2015). This parallel may indicate that the reduced spine-print identifiability, relative to brain fingerprinting, reflects functional characteristics of sensorimotor systems more generally.

Future work should aim to explore the stability of this spine-print across sessions to determine whether it reflects a short-term phenomenon or a more persistent individual trait. However, this remains technically challenging. The spinal cord’s curvature is highly influenced by participant positioning and can vary significantly across sessions, posing challenges for accurate between-session image co-registration. To address this, an informative next step would involve scanning the same individual in two consecutive sessions with only a brief pause in between, during which the participant is temporarily removed from the scanner but remains positioned on the table—compared to a condition where the participant is repositioned entirely. This approach would allow for a more controlled assessment of acquisition-related variability, disentangled from repositioning effects.

Additionally, our findings highlight the potential role of the brain in shaping spinal cord activity. Given the observed interactions, future research should further investigate the dependency and directionality of brain–spine functional connectivity, which may yield new insights into hierarchical control mechanisms within the central nervous system.

## Supplementary Material

Supplement 1

## Data Availability

*Dataset 2* is publicly available, while *Dataset 1* and *3* are available upon reasonable request to Dr. Robert Barry and Prof. Julien Doyon, respectively. Code is publically available on the GitHub repository: https://github.com/MIPLabCH/SpinePrint.

## LIST OF ABBREVIATIONS

FC: functional connectivity
ROI: region of interest
ICC: inter cluster coefficient
Idiff: identifiability score
*dh*: dorsal horns
*Iz*: intermediate zone
*vh*: ventral horns
*cst*: cortico-spinal tract
*fc*: fasciculus cuneatus
*fg*: fasciculus gracilis
*sl*: spinal lemniscus
VIS: visual network
SM: somatomotor network
DA: dorsal-attention network
VA: ventral-attention network
L: limbic network
FP: fronto-parietal network
DMN: default-mode network
GM: gray matter
WM: white matter
